# Causal Inference of Body Ownership in the Posterior Parietal Cortex

**DOI:** 10.1523/JNEUROSCI.0656-22.2022

**Published:** 2022-09-14

**Authors:** Marie Chancel, Heather Iriye, H. Henrik Ehrsson

**Affiliations:** Department of Neuroscience, Karolinska Institutet, SE-17177 Stockholm, Sweden

**Keywords:** Bayesian causal inference, functional MRI, multisensory integration, neuroimaging, psychophysics, rubber hand illusion, multisensory integration, psychophysics, body perception

## Abstract

How do we come to sense that a hand in view belongs to our own body or not? Previous studies have suggested that the integration of vision and somatosensation in the frontoparietal areas plays a critical role in the sense of body ownership (i.e., the multisensory perception of limbs and body parts as our own). However, little is known about how these areas implement the multisensory integration process at the computational level and whether activity predicts illusion elicitation in individual participants on a trial-by-trial basis. To address these questions, we used functional magnetic resonance imaging and a rubber hand illusion-detection task and fitted the registered neural responses to a Bayesian causal inference model of body ownership. Thirty healthy human participants (male and female) performed 12 s trials with varying degrees of asynchronously delivered visual and tactile stimuli of a rubber hand (in view) and a (hidden) real hand. After the 12 s period, participants had to judge whether the rubber hand felt like their own. As hypothesized, activity in the premotor and posterior parietal cortices was related to illusion elicitation at the level of individual participants and trials. Importantly, activity in the posterior parietal cortex fit the predicted probability of illusion emergence of the Bayesian causal inference model based on each participant's behavioral response profile. Our findings suggest an important role for the posterior parietal cortex in implementing Bayesian causal inference of body ownership and reveal how trial-by-trial variations in neural signatures of multisensory integration relate to the elicitation of the rubber hand illusion.

**SIGNIFICANCE STATEMENT** How does the brain create a coherent perceptual experience of one's own body based on information from the different senses? We examined how the likelihood of eliciting a classical bodily illusion that depends on vision and touch—the rubber hand illusion—is related to neural activity measured by functional magnetic resonance imaging. We found that trial-by-trial variations in the neural signal in the posterior parietal cortex, a well known center for sensory integration, fitted a statistical function that describes how likely it is that the brain infers that a rubber hand is one's own given the available visual and tactile evidence. Thus, probabilistic analysis of sensory information in the parietal lobe underlies our unitary sense of bodily self.

## Introduction

Body ownership, the sense of our body as our own (Ehrsson, 2020), is a fundamental aspect of the human mind that creates a boundary between oneself and the external world that is critical for self-awareness and effective goal-directed and defensive actions (Botvinick and Cohen, 1998; Graziano et al., 2000; Ehrsson et al., 2004; Fang et al., 2019). Accordingly, disturbances in own-body perception are an important topic in medicine and psychiatry (Brugger and Lenggenhager, 2014; Keizer et al., 2014; Jenkinson et al., 2018; Costantini et al., 2020; Garbarini et al., 2020; Saetta et al., 2020). The rubber hand illusion (RHI) is the most widely used experimental paradigm to investigate the perceptual processes underlying own-body perception in healthy participants. It consists of eliciting illusory ownership toward a human-like model hand through correlated visual, tactile, proprioceptive, and other body-related sensory signals (Botvinick and Cohen, 1998; Ehrsson, 2020). By synchronously stroking the rubber hand, in full view of the participant, and the participant's corresponding hidden real hand, most participants feel that the fake hand is their own. Behavioral RHI studies have shown that the integration of visual and somatosensory signals plays a critical role in subjective changes of body ownership (Ehrsson, 2012, 2020).

Previous fMRI studies have identified activity in brain regions associated with multisensory integration during the RHI, including the posterior parietal cortex (PPC), premotor cortex, lateral occipital cortex, cerebellum, and putamen (Ehrsson et al., 2004; Ehrsson, 2005; Makin et al., 2007; Gentile et al., 2013; Bekrater-Bodmann et al., 2014; Limanowski and Blankenburg, 2016b). In these studies, an illusion condition with synchronous and spatially congruent multisensory stimulation is compared with various control conditions that violate temporal and/or spatial principles of multisensory integration. Resulting activations of the ventral premotor cortex (PMv) and the intraparietal sulcus (IPS) have attracted particular interest because they integrate visual, tactile, and proprioceptive signals from the upper limb (Lloyd et al., 2003; Makin et al., 2007; Gentile et al., 2011, 2013). Moreover, single and multiunit recordings in nonhuman primates have identified groups of neurons in these regions that could reflect multisensory integration (Duhamel et al., 1998; Graziano, 1999; Graziano et al., 2000; Avillac et al., 2007; Fang et al., 2019). However, little is known about how these areas implement the multisensory integration underlying the RHI at the computational level and how the neural signature of multisensory integration during illusion elicitation relates to body ownership perception on a trial-by-trial basis.

To address these questions, we used an fMRI approach based on a detection-like psychophysics task and computational modeling. While fMRI scans were registered, healthy participants repeatedly performed 12 s trials of visuotactile stimulation delivered to the rubber hand (in view) and their real hand (out of view) with subtle variations in the degree of asynchrony (0, ±150, ±300, and ±500 ms). After each trial, participants judged whether the rubber hand felt like their own hand or not (yes/no detection-like judgments). Responses were fitted by a Bayesian causal inference (BCI) model (Chancel et al., 2021a) that predicted the individual probability of the emergence of the RHI based on probabilistic computational principles of multisensory perception (Körding et al., 2007). The causal inference model describes how the brain decides whether the visual and somatosensory signals are integrated (eliciting the illusion) or segregated (no illusion) based on the temporal correspondences of multisensory stimulations and prior knowledge (Samad et al., 2015; Ehrsson and Chancel, 2019; Fang et al., 2019; Chancel et al., 2021a). By analyzing how trial-by-trial variations in BOLD responses relate to predictions of the BCI model based on each participant's behavioral response profile, we sought to test the hypothesis that PPC and premotor cortex implement the causal inference of body ownership. Moreover, to clarify the relationship between the subjective illusion and neural responses at the level of individual participants and trials, we contrasted trials when the illusion was detected with trials when it was not. We hypothesized that BOLD signals in the above-mentioned frontoparietal areas would reflect the subjective illusion in this trial-by-trial perception-based approach.

## Materials and Methods

### Participants

Forty-three healthy, naive participants were recruited for this experiment (21 females; mean age, 26.4 ± 6 years). The predetermined sample size was 30 fully completed experiments with quality data, and we kept recruiting participants until we reached this number before starting the statistical analysis of the fMRI data. This sample size of 30 was chosen according to what is usually found in the RHI neuroimaging literature (Gentile et al., 2013; Bekrater-Bodmann et al., 2014; Limanowski and Blankenburg, 2018). All volunteers provided their written informed consent before their participation. Five participants did not meet the inclusion criteria (see below). Three participants could not complete the scanning session because of technical failure. All participants received monetary compensation for their participation. After preprocessing of the fMRI scans, five participants were excluded because of motion artifacts. As a result, imaging data from 30 participants were fully analyzed in this study (13 females; mean age, 26.1 ± 6 years). All experiments were approved by the Swedish Ethics Review Authority (Ethics #2018/471–31/2).

### Experimental setup

During the experiment, participants lay inside the fMRI scanner with their head tilted slightly forward (∼30°). The participant's right hand lay next to their body, palm down, on a flat supportive surface, tilted upward (∼30°). We chose this position because it would allow participants to lie down comfortably and still have a clear view of their right hand in direct sight. Via an MR-compatible 3D headset (NordicNeuroLab; FOV, 30° horizontal × 23° vertical; resolution, 800 × 600), the participant saw a cosmetic prosthesis of a right hand filled with plaster (hereafter referred to as the “rubber hand”) in the same anatomic position and a similar location as their right hand. The real hand was closer to the horizontal axis (20°) than the rubber hand in view (40°), reproducing the classical proprioceptive mismatch of the rubber hand illusion paradigm with a vertical arrangement of rubber hand on top of the real hand (Ehrsson et al., 2004). A 3D video of the rubber hand being touched by the same MR-compatible robot that was used to touch the participants' real hand during the experiment was prerecorded and used as a visual stimulus. Great care was taken to ensure that each participant's position, each participant's real position of his or her right hand, and the robot position in the scanner matched the position in which this 3D video was recorded. The participants' body and the body in the video were covered by the same thick black cover to maximize visual similarity between the scanning scene and the recorded visual scene. We used a video recording instead of live stimulations to control the exact relative timing of the visual and tactile stimuli, which is important for the current psychophysics approach; moreover, we only needed one robot to stimulate the real hand instead of two robots, and only one robot was fitted in the constrained space of the tunnel of the MR scanner.

**Figure 1.**
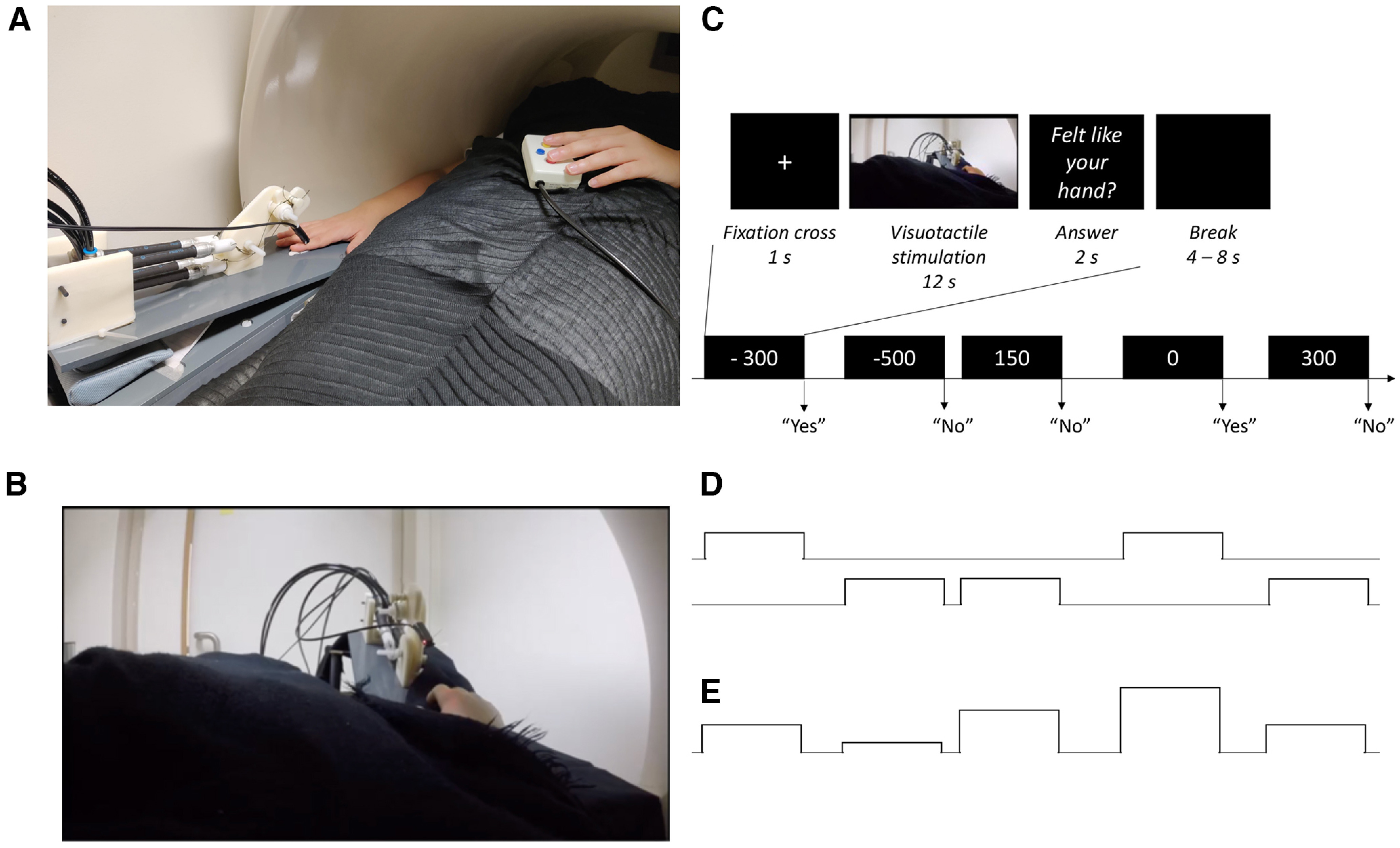
Experimental setup and procedure. ***A***, The participant's right arm and hand lay next to their body in a relaxed position, palm down, on a flat support, tilted upward (∼20°), and a robot arm applied tactile stimuli (taps) to the index fingers of the rubber hand in the video and to the participant's real hand during the experiment. ***B***, A 3D video that was prerecorded was used as a visual stimulus in the experiment showing the rubber hand (∼40°) being touched by the same MR-compatible robot that was used to touch the participants' real hands during the experiment. ***C***, All trials followed the same sequence: after the presentation of a fixation cross for 1 s, participants saw the rubber hand being touched in the head-mounted display while their hand was touched by the robot, synchronously or not. This 12 s visuotactile stimulation was followed by a 2 s display of the question “[did the rubber hand] feel like [it was] your [own] hand?” to which participants answered “yes” or “no” by pressing the corresponding key with their left hands. This schematic representation of the procedure shows an example of a sequence of five consecutive trials with five of the seven different asynchrony conditions (−300, −500, +150, 0, and +300 ms; the + 500 and –150 ms conditions are not shown in this example). ***D***, The collected yes/no judgments (here, a theoretical example) were used in an fMRI analysis to define a regressor at the first level for the trials eliciting the RHI (represented by the top boxcar) and one for the trials not eliciting any illusion (represented by the bottom boxcar). ***E***, These participants' answers were also fitted individually in our BCI model to estimate the probability of emergence of the RHI for a given asynchrony for each participant. These estimates were used to build a parametric modulation regressor at the first level to test for brain regions showing a relationship between the predictions of the BCI model and the strength of neural response across the different asynchronies tested.

A robot arm (designed by Martti Mercurio and author M.C.) applied tactile stimuli (taps) to the index fingers of the rubber hand in the video and to the participant's real hand during the experiment (Fig. 1). The robot arm was made of the following three parts: two 10-cm-long, 5-cm-wide plastic pieces, and a plastic slab (15 × 55 cm) as a support. The joint between the two plastic pieces and the one between the proximal piece and the support were powered by a pneumatic muscle (catalog #DMSP-10-100N-RM-CM, Festo) connected to four pneumatic valves outside of the scanner room (catalog #VPPX-6L-L-1-G18-0L10H-S1, Festo). The distal plastic piece ended with a plastic rod (diameter, 10 mm) that was used to touch the rubber hand in the video and the participant's real hand during the experiment. Finally, the participants' left hands rested on their left hips. In their left hands, the participants held a two-key keyboard that they used to respond during the task.

### Procedure

In each trial, participants were to decide whether the rubber hand felt like it was their own hand or not (i.e., determine whether they felt the key phenomenological aspect of the rubber hand illusion; Botvinick and Cohen, 1998; Ehrsson et al., 2004; Longo et al., 2008). Each trial followed the same sequence: the robot repeatedly tapped the index finger the participant's hand six times each for a total period of 12 s in five different locations (the “stimulation phase”), just proximal to the nail on the distal phalanx, on the distal interphalangeal joint, on the middle phalanx, on the proximal interphalangeal joint, and on the proximal phalanx (Chancel et al., 2021a, stimulation protocol). In addition to this tactile stimulation, the participant saw the same sequence of touches applied to the rubber hand via the head-mounted display. The participant was instructed to focus their gaze on the rubber hand. Then, the robot stopped, and a black screen was displayed in the headset. The question “[Did the rubber hand] feel like [it was] your [own] hand?” appeared for 2 s (the question was shortened for display purposes, but the participants were given the fully written question in the instructions before the experiment began); the participant had to press a key with his or her left index finger to answer “yes” (the rubber hand felt like it was my own hand) or with their middle finger to answer “no” (the rubber hand did not feel like it was my own hand). A period of 12 s was chosen in line with a previous rubber hand illusion-psychophysics study (Chancel and Ehrsson, 2020) and because the illusion is, in most cases, elicited within ∼10 s of synchronous visuotactile stimulation on average in individuals susceptible to the illusion (Ehrsson et al., 2004; Lloyd, 2007; Guterstam et al., 2013). Different locations on the finger were chosen to prevent irritation of the skin during the repeated stimulation with many trials (Chancel and Ehrsson, 2020; Chancel et al., 2021a) and in line with earlier studies that often stimulate different parts of the hand and fingers to elicit the rubber hand illusion (Guterstam et al., 2011). After the 2 s period during which the participants pressed the key corresponding to their answer (yes or no), the question disappeared, leaving only a black screen that was displayed for 6 s on average (jitter from 4 to 8 s). A white fixation cross appeared for 1 s to inform the participant that a new trial was about to start.

We manipulated the degree of asynchrony between the seen and felt taps in seven different steps (“asynchrony conditions”). The video of the touches applied to the rubber hand could be synchronized with the sequence of touches on the participant's real hand (synchronous condition), or the onset of the video could be delayed or advanced by 150, 300, or 500 ms. In the rest of this article, negative values of asynchrony (−150, −300, and −500 ms) mean that the rubber hand was touched first, and positive values of asynchrony (+150, +300, and +500 ms) mean that the participants' hands were touched first. The seven levels of asynchrony appeared with equal frequencies in a pseudorandom order (i.e., no condition was repeated more than twice in a row). Each condition was repeated 20 times, leading to a total of 140 judgments per participant. These trials were pseudorandomly divided into five functional runs, each lasting 9 min and 48 s.

### Inclusion test

We wanted to ensure that participants understood the task correctly and were familiarized with the different parts of the setup and the task to be able to perform it well later in the MR environment. Therefore, they came to the MR center a first time to be tested in a mock MRI scanner (but without scanner noise). The conditions were identical to those of the main experiment, but no neuroimaging data were acquired. Participants received the equivalent of one functional run (10 min, 28 trials, four repetitions per asynchrony). We were ultimately interested in contrasting the trials for which the participant replied “yes” and those for which they replied “no” to the illusion question (see above). Thus, we chose the following inclusion criteria: at least 2 of the 28 responses given by the participant during this mock scanner testing needed to be different (i.e., a participant who always gave the same answer; always yes or always no) was excluded from the actual scanning session (because their data cannot be modeled with the current analysis approaches in any meaningful way). Of the 43 participants we tested in the mock scanner, 5 participants did not meet this inclusion criterion (3 answered “yes” to all trials, and the other 2 answered “no” to all trials). All 38 other participants returned on another day to be included in the main experiment (of which 8 participants were later excluded, as mentioned above, because of technical failure and excessive head movements; see further below).

### MR acquisition parameters

MRI data were acquired using a scanner (TIM Trio 3T, Siemens) equipped with a 16-channel head coil. Gradient echo T2*-weighted EPIs with BOLD contrast were used as an index of brain activity (Logothetis et al., 2001). A functional image volume was composed of 42 continuous near-axial slices of 3 mm thickness (interslice gap, 0.5 mm), which ensured that the whole brain was within the FOV (matrix, 96 × 96; in-plane resolution, 3.0 × 3.0 mm; TE = 30 ms). One complete volume was collected every 2.2 s (TR = 2204 ms). A total of 1280 functional volumes were collected for each participant; volume acquisition was equally divided into five sessions (i.e., functional runs). An initial baseline of 15 s and a final baseline of 15 s were included in each of these sessions for all experiments. The first five volumes of each session were automatically discarded to account for non-steady-state magnetization. Triggers were collected for each new volume acquisition to ensure correct timing among the acquired scan, the robot movements, and the 3D videos. To facilitate the anatomic localization of statistically significant activations, a high-resolution structural image was acquired for each participant after the first three functional runs of the experiment (3DT1 sequence: voxel size = 1 × 1 × 1 mm; FOV = 255 × 204 mm; 176 slices; TI = 450 ms, TE = 3.18 ms, TR = 8.16 ms; flip angle = 12°).

### Behavioral data analysis

The percentage of “yes” answers per asynchrony and per participant was calculated. The emergence of the rubber hand illusion is driven by the integration of visual and tactile signals; and in the current paradigm, the smaller the asynchrony was during a given trial, the greater the likelihood that the illusion would be elicited in that trial. In a previous behavioral study, we designed a model in which the observer performs Bayesian causal inference that successfully describes this integration (Chancel et al., 2021a). We used the same BCI model to be fitted to the participants' answers in the present study and the same fitting procedure. Below, we briefly describe our modeling approach, and more details can be found in the study by Chancel et al. (2021a).

Bayesian inference is based on a generative model, which is a statistical model of the world that the observer believes gives rise to observations. By “inverting” this model for a given set of observations, the observer can make an “educated guess” about a hidden state. In our case, the model contained the following three variables: the causal structure category C, the tested asynchrony s, and the measurement of this asynchrony by the participant x. Although the true frequency of synchronous stimulation (*C* = 1) was 1 of 7 = 0.14, we allowed it to be a free parameter, which we denoted as *p*_same_. Next, we assumed that for the observer, when *C* = 1, the asynchrony *s* was always 0. When *C* = 2, the true asynchrony took one of several discrete values; we did not presuppose that the observer knew these values or their probabilities but instead assumed that asynchrony was normally distributed with the correct SD $\sigma_{S}$ of 348 ms (i.e., the true SD of the stimuli used in this experiment). In other words, $p\left(s|C=2\right)=N\left(s;0,\sigma_{s}^{2}\right)$. Next, we assumed that the observer made a noisy measurement, *x*, of the asynchrony. We made the standard assumption (inspired by the central limit theorem) that this noise adhered to the following normal distribution:

$$p\left(x|s\right)=N\left(x;s,\sigma^{2}\right),$$

where the variance depends on the sensory noise for a given trial.

From this generative model, we turned to inference. Visual and tactile inputs are to be integrated, leading to the emergence of the rubber hand illusion if the observer infers a common cause (C=1) for both sensory inputs. On a given trial, the model observer uses x to infer the category C. Specifically, the model observer computes the posterior probabilities of both categories, p(C=1|x) and p(C=2|x) (i.e., the belief that the category was C). Then, the observer would report “yes, it felt like the rubber hand was my own hand” if the former probability were higher, or, in other words, when d>0, as in the following:

$$d=\text{log}\frac{p\left(C=1|x\right)}{p\left(C=2|x\right)}.$$

The decision rule *d* > 0 is thus equivalent to the following:

$$|x|<\sqrt{K},$$

where

$$K=\frac{\sigma^{2}(\sigma_{s}^{2}+\sigma^{2})}{\sigma_{s}^{2}}\left(2\text{log}\frac{p_{\text{same}}}{1-p_{\text{same}}}+\text{log}\frac{\sigma_{s}^{2}+\sigma^{2}}{\sigma^{2}}\right),$$

where σ is the sensory noise level of the trial under consideration. As a consequence, the decision criterion changes as a function of the sensory noise affecting the observer's measurement. The output of the BCI model is the probability of the observer reporting the visual and tactile inputs as emerging from the same source when presented with a specific asynchrony value s, as follows:

$$p\left(\hat{C}=1|s\right)=0.5\lambda +(1-\lambda )\left(\Phi \left(s;k,\sigma^{2}\right)-\Phi \left(s;-k,\sigma^{2}\right)\right).$$

Here, the additional parameter λ reflects the probability of the observer lapsing (i.e., randomly guessing). This equation is a prediction of the observer's response probabilities and can thus be fitted to a participant's behavioral responses. Thus, our BCI model has five free parameters: $p_{\text{same}}$, the prior probability of a common cause for vision and touch, independent of any sensory stimulation; σ, the noise impacting the measurement x; and λ, a lapse rate to account for random guesses and unintended responses. We assumed a value of 348 ms for $\sigma_{S}$ (i.e., $\sigma_{S}$ is equal to the actual SD of the asynchronies used in the experiment). Model fitting was performed using maximum-likelihood estimation implemented in MATLAB (MathWorks). We used the Bayesian Adaptive Directed Search algorithm (Acerbi and Ma, 2017), each using 100 different initial parameter combinations per participant. The overall goodness of fit was assessed using the coefficient of determination *R*^2^ (Nagelkerke, 1991) defined as follows:

$$R^{2}=1-\text{exp}\left(-\frac{2}{n}\left(\text{log}L\left(M\right)-\text{log}L\left(M_{0}\right)\right)\right),$$

where logL(M) and log$L\left(M_{0}\right)$ denote the log-likelihoods of the fitted and null models, respectively, and *n* is the number of data points. For the null model, we assumed that an observer randomly chose one of the two response options (i.e., we assumed a discrete uniform distribution with a probability of 0.5). As in our case, the models' responses were discretized to relate them to the two discrete response options; the coefficient of determination was divided by the maximum coefficient (Nagelkerke, 1991), defined as follows:

$$\max\left(R^{2}\right)=1-\exp\left(\frac{2}{n}\text{log}L\left(M_{0}\right)\right).$$

### MR data analysis

#### Preprocessing.

All fMRI data were screened for potential motion and physiological artifacts using the ArtRepair toolbox (Mazaika et al., 2009). The fMRI data were also corrected for field map distortion using a script created by MR Center (Karolinska Institutet) physicist Rouslan Sitnikov using FSL (FMRIB software library). The functional imaging data then underwent a series of standard preprocessing steps using Statistical Parametric Mapping 12 software [SPM12, Wellcome Trust Center for Neuroimaging (https://www.fil.ion.ucl.ac.uk/spm/)] before all successive analyses. The functional volumes were motion corrected with respect to the first volume of each series, corrected for slice-timing errors, and coregistered to the high-resolution structural image. The latter was segmented into gray matter, white matter, and CSF partitions, and was normalized to the standard MNI space. The same transformation was then applied to all functional images, which were spatially smoothed with a 6 mm FWHM Gaussian kernel. Volumes with excessive head motion were interpolated using the ArtRepair toolbox (movement threshold, 0.5; rotation threshold, 0.02). If > 10% of volumes had to be motion corrected in a particular participant, that participant's entire data were excluded from further analysis to ensure that all data that went into the main analysis were of high quality (*N* = 5).

#### Illusion-detection contrast.

We fitted a general linear model to the data for each individual participant. We defined boxcar regressors for the two conditions of interest with respect to RHI detection (i.e., the 12 s visuotactile stimulation that proceeded the participant's yes/no response to the following illusion question: Did the rubber hand feel like it was your own hand?). These 12 s blocks were convolved with the standard hemodynamic response function modeled in SPM12. A separate regressor of no interest was also included to model the 2 s period after the participants pressed the button on the response keyboard (and this regressor was also convolved with the standard hemodynamic response function). Linear contrasts of interest were defined for each participant as appropriate combinations of the model parameters and exported to a second-level random-effects analysis. For this second-level analysis, we contrasted the 12 s stimulation trials that led to a “yes” answer to the trials that led to a “no” answer (the movement-related regressor of no interest was not used in the analysis but simply served to model out motor-related activation).

#### BCI model fitting and parametric modulation.

To fit the trial-by-trial BOLD modulations across the different levels of asynchrony to the BCI model, we defined a parametric contrast at the first level. All the trials were modeled with a unique regressor. However, with this regressor, the boxcar function representing each trial was modulated by the probability of emergence of the rubber hand illusion predicted by our BCI model corresponding to the given visuotactile asynchrony. For each participant, we used the results of the BCI model fitting on their individual responses; therefore, the modulators included in the regressor were specific to each participant. Note also that in SPM parametric modulators are automatically orthogonalized from the main effect regressor (i.e., a regressor representing the effect of the visuotactile stimulation without any modulation). Thus, the parametric modulations regressor identifies activity that fits the predictions of the BCI model over and above the neural responses triggered by the visuotactile stimulation. Once again, a regressor of no interest was also included to account for the participants' finger movements when they answered by pressing a button on the response keyboard. These individually modulated images were then evaluated on the second level using a one-sample *t* test against 0.

#### Statistical approach: voxel-based whole-brain analysis, neuroanatomical hypotheses, and corrections for multiple comparisons.

We analyzed the activity of all voxels in the whole brain using the SPM approach. In line with common practice, the resulting activation maps were first thresholded using a voxelwise threshold of *p* < 0.001 and a minimum cluster size of 10 voxels. These whole-brain “uncorrected” activation maps are seen in Extended Data Tables 2-1 and 3-1 and are depicted in the figures for purely descriptive purposes (to illustrate the anatomic specificity and topography of the activations and to facilitate future meta-analyses and neuroanatomical hypothesis generation). These activation maps were also projected onto the mean anatomic image from our participant pool (see Fig. 6). For statistical inference, we corrected for multiple comparisons using familywise error (FWE) correction *p* < 0.05 across two complementary approaches.

**Table 1.** Significant activations in the RHI illusion-detection analysis (“yes” trials vs “no” trials concerning the question: Did the rubber hand feel like it was your own hand?)

MNI coordinates (mm)			Cluster level		Peak level	Anatomical region (functional area)
*x*	*y*	*z*	Cluster size (*k*)	*p* (FWE)	*z*	
−32	−80	30	693	0.00	5.09	L IPS
−24	22	50	216	0.01	4.54	L superior frontal sulcus (dlPFC)
−46	−70	−8	141	0.05	4.38	LOC
−20	−48	50	178	0.02	4.10	L superior parietal sulcus
−2	−48	10	313	0.00	4.04	L cingulate sulcus/retrosplenial cortex
−54	4	28		0.006	3.94	L precentral gyrus (PMv)*
−30	−50	54		0.013	3.71	L IPS*

L, Left; LOC, left inferior occipital sulcus.

*MNI coordinates (*x*, *y*, *z*) and *p*-values are based on whole-brain or small-volume correction.

First, for the cortices lining the intraparietal sulcus in the PPC and the ventral premotor cortex, where we had a strong a priori hypothesis based on the previous fMRI literature, we used the so-called “small-volume correction” (SVcorr) procedure, where we corrected for the number of voxels in a 10-mm-radius sphere around activation peaks from a previous RHI study (Ehrsson et al., 2004: left PMv: *x* = −54, *y* = −2, *z* = 28; left IPS: *x* = −33, *y* = −51, *z* = 63) based on an FWE correction of peak height (SVcorr). These specific PMv and IPS activations have been replicated in similar fMRI studies (Ehrsson, 2005; Gentile et al., 2013; Guterstam et al., 2015), and two meta-analyses of body ownership neuroimaging studies have shown consistent activation of these regions (Grivaz et al., 2017; Seghezzi et al., 2019). Moreover, the premotor cortex in nonprimates has been implicated in causal inference of limb embodiment (Fang et al., 2019) and human neuroimaging studies on audiovisual integration and theoretical considerations that point toward the PPC as a key region for implementing casual inference of multisensory integration (Rohe and Noppeney, 2015, 2016; Rohe et al., 2019). Note that other regions, such as the putamen, lateral occipital cortex, insula, and cerebellum, have been suggested to play important roles in the RHI, but, given our novel analysis approach, we only used the small-volume correction approach for two regions in this study to reduce the risk of type II errors.

Second, we searched for activations in the whole brain, and, for this more explorative approach, we corrected for the number of voxels in the whole brain. Here, we used a threshold of *p* < 0.05 after FWE correction for the whole brain space (WBcorr) based on a cluster size test. Note that this approach is very conservative and there is a substantial risk of type I errors.

All reported coordinates are in MNI space, and we also report cluster sizes (*k*) and *z* scores for all peaks. Note that for visualization purposes only, all activation maps are displayed in the figures at a threshold of *p* < 0.001 (uncorrected), but the significant activations that survive correction for multiple comparisons are always clearly labeled and circled in the figures.

For anatomic localization of the activations, the activation peaks were overlaid on the average anatomic MRI image for all participants and referred to macroanatomical landmarks (sulci and gyri) using the terminology from the Duvernoy and Parratte brain atlas (Duvernoy, 1999).

## Results

### Behavioral results

**Figure 2.**
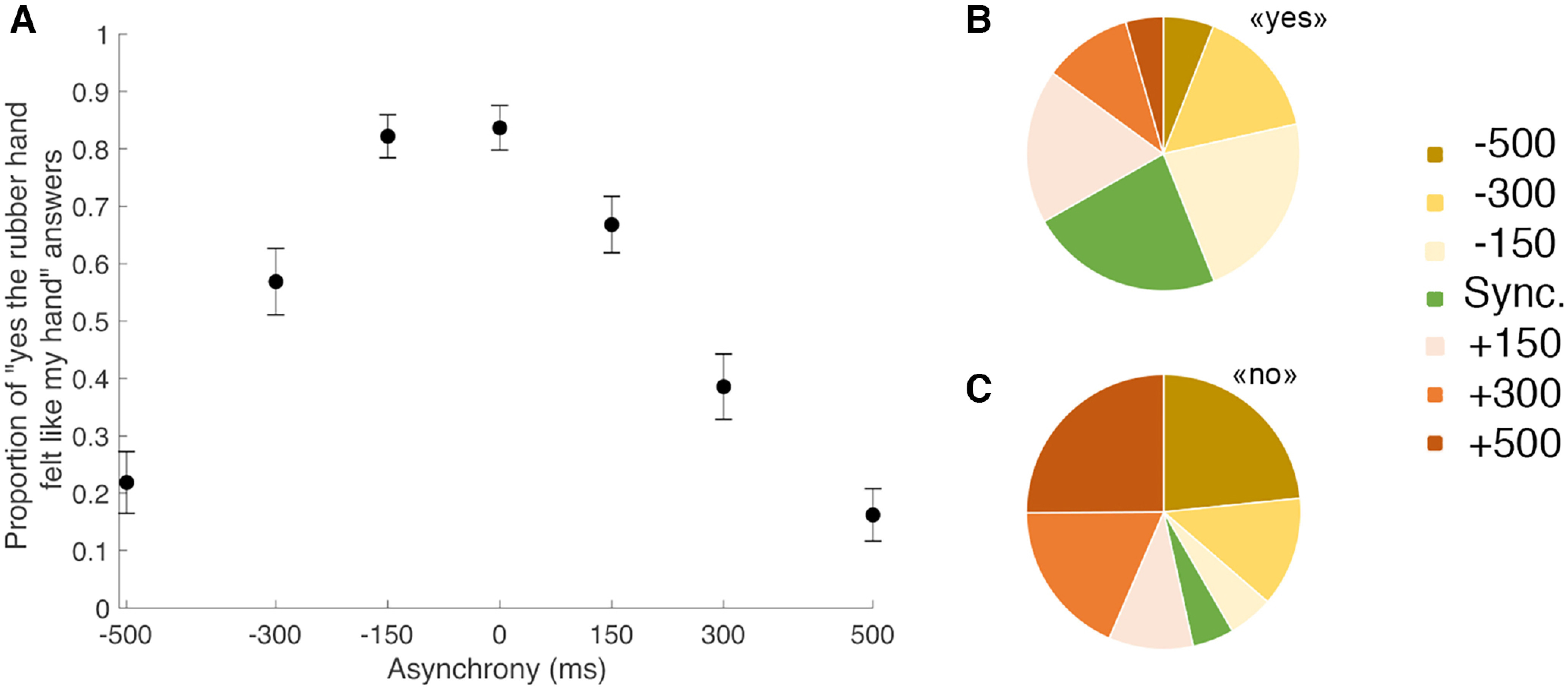
RHI elicited under different levels of asynchrony. ***A***, The black dots represent the reported proportion of rubber hand illusion detection (i.e., responding “yes” to the statement “[did the rubber hand] felt like [it was] your [own] hand”; mean ± SEM) for each of the seven asynchrony conditions (−500, −300, 0, +150, +300, and +500 ms). In the synchronous condition, the participants reported perceiving the rubber hand like their own hand in 84 ± 4% (mean ± SEM) of the 20 trials when the visual and tactile stimulations were presented simultaneously (no asynchrony). Moreover, for every participant, increasing the asynchrony between the seen and felt touches decreased the prevalence of the illusion in a graded fashion: when the rubber hand was touched 500 ms before or after the real hand was touched, the illusion was reported only in 22 ± 5% and 16 ± 5% of the 20 trials, respectively. ***B***, Repartition of the trials in which the RHI was detected by asynchrony conditions (color coded). For example, synchronous visuotactile stimulation (0 ms condition) accounted for 23% of illusion detections, and consequently, 77% of the “yes” trial responses occurred following stimulation with varying degrees of asynchrony. ***C***, Repartition of the “no” trials when the participants judged that the RHI had not been experienced (responding “no” to the statement above). Synchronous visuotactile stimulation (0 ms condition) accounted for 5% of the unsuccessful RHI fixations across all trials, while trials with maximum asynchrony (±500 ms) accounted for 48% of the total number of “no” trials across all conditions.

**Figure 3.**
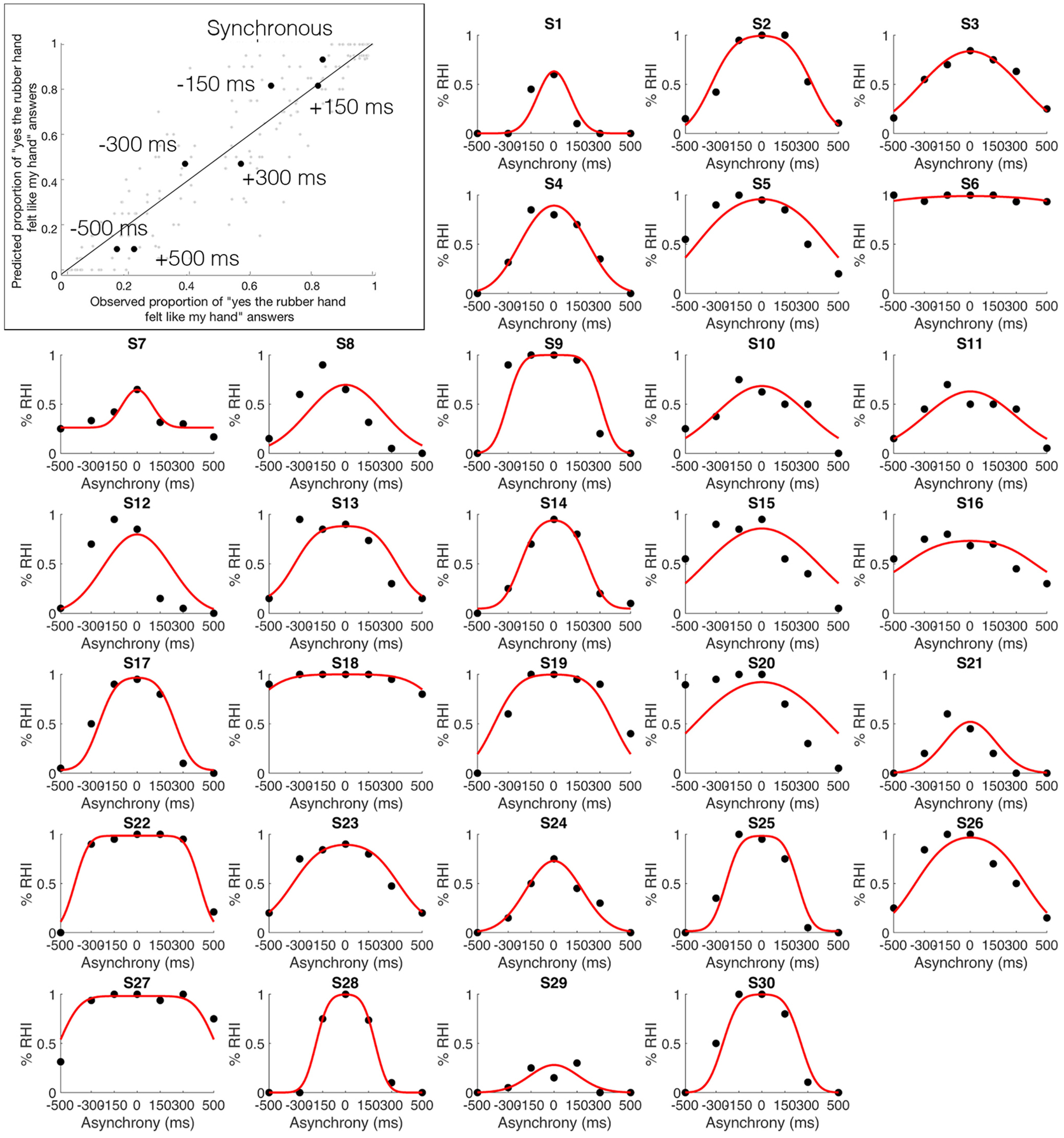
Observed and predicted probability of the emergence of the RHI. Top left corner, Mean observed probability of the emergence of the RHI (percentage “yes” judgments; *x*-axis) plotted against the probability of the emergence of the RHI predicted by the BCI model (*y*-axis) for the seven different asynchrony conditions (black dots; color-coded conditions). ***S1–S30***, The proportion of RHI elicitations reported by each of the 30 individual participants (*x*-axes) for each level of asynchrony (*y*-axes) is shown (black dots), as is the distribution predicted by the BCI model (red curve; Extended Data Table 1-1, information about the estimated model parameters).

As described above, participants performed a detection-like task on the ownership they felt toward the rubber hand; the tactile stimulation they felt on their hidden real hand was synchronized with the touches they saw on the rubber hand or systematically delayed or advanced (in seven steps). For each degree of asynchrony, the percentage of trials for which the participants felt like the rubber hand was theirs was determined (Fig. 2). The rubber hand illusion was successfully modulated as we had expected, in line with our gradual manipulation of asynchrony (Chancel and Ehrsson, 2020; Chancel et al., 2021a,b). Moreover, the repartition of answers across different levels of asynchrony and different trials revealed a sufficient degree of individual variability (Fig. 3) for our computational modeling and trial-by-trial and model-based fMRI analyses to work.

10.1523/JNEUROSCI.0656-22.2022.t1-1 Table 1-1 Individual estimated parameters for the BCI model. Download Table 1-1, DOCX file.

### BCI modeling of behavioral results

The probability of emergence of the rubber hand illusion predicted by the BCI model (Chancel et al., 2021a) fit well the observed probability of emergence of the rubber hand illusion (mean ± SEM: *R*^2^ = 0.60 ± 0.04; Fig. 3, top left). Thus, the model captured the individual participant's perceptual ownership decision in a graded quantitative manner; the model also considered that this perception varied among participants (Fig. 3, individual plots). The precise probability of emergence of the RHI estimated by our BCI model for each visuotactile asynchrony differed for every participant; notably, it is these participant-specific estimates that we used in the parametric modulation fMRI analysis to look for brain responses that indicated causal inference of body ownership. Details about the corresponding estimated model parameters can be found in Extended Data Table 1-1.

### fMRI analyses

#### Illusion-detection contrast

**Figure 4.**
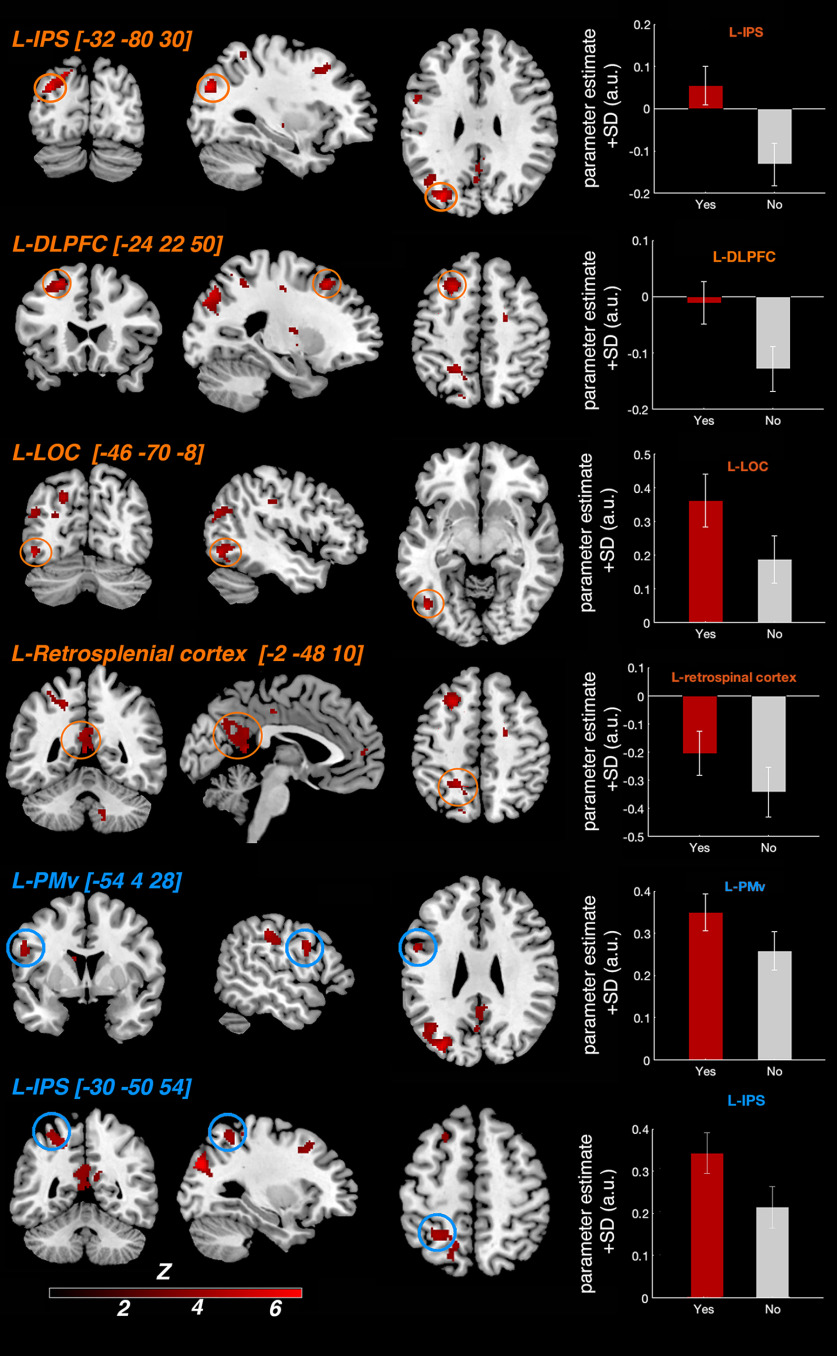
Activations related to RHI detection. Increased BOLD signal when contrasting trials in which visuotactile stimulation led to participants answering “yes” to the illusion question (did the rubber hand felt like it was your own hand?) compared with stimulations for which participants answered “no” to this question across all levels of asynchrony/synchrony. For display purposes only, the activation map is displayed at a threshold of *p* < 0.001 (uncorrected for multiple comparisons, cluster threshold: 10 voxels), projected on a single-subject T1 MNI template (Fig. 6, presentation on the participants' mean structural MRI). The six highlighted activations were all significant (*p* < 0.05) after correction for multiple comparisons. Areas circled in orange survived whole-brain correction, and areas circled in blue survived small-volume correction based on a priori anatomic hypotheses (Table 1). The right panels show the BOLD signal (contrast estimates extracted from a sphere with a 5 mm radius center on the peak activation) from the six regions in question for the “yes” trials (red) and “no” trials (gray; compared with the baseline) to illustrate the effect sizes for purely descriptive purposes. LOC, Lateral occipital cortex.

We first looked for neural responses related to the elicitation of illusory rubber hand ownership in each participant across the different asynchrony levels on a trial-by-trial basis. Namely, the difference in BOLD signal between trials when visuotactile stimulation led to the participant judging “yes [the rubber hand felt like it was my own hand]” compared with trials when stimulation led to the participants answering “no” to this question, regardless of the degree of visuotactile asynchrony or synchrony (Fig. 4, Table 1, Extended Data Table 2-1).

10.1523/JNEUROSCI.0656-22.2022.t2-1 Table 2-1 Result table for the answer-based contrasts (*p* < 0.001 uncorrected; extent threshold = 10 voxels), localization is based on peak activity. Download Table 2-1, DOCX file.

As hypothesized, this analysis revealed significant BOLD responses in the PPC and the premotor cortex [Table 1, Fig. 4 (see also Fig. 6*A*)]. In the PPC, we observed significant activations in cortices lining the left intraparietal sulcus (*x* = −20; *y* = −48; *z* = 50; *k* = 178 voxels; Z = 4.10; *p* < 0.001, WBcorr) and two foci in the left IPS (*x* = −32; *y* = −80; *z* = 30; *k* = 693 voxels; Z = 5.09; *p* = 0.02, WBcorr; *x* = −30, *y* = −50, *z* = 54; Z = 3.71; *p* = 0.013, SVcorr, Z = 3.71). In the premotor cortex, we observed significant activation located in the left precentral gyrus corresponding to the PMv (*x* = −54, *y* = 4, *z* = 28; Z = 3.94; *p* = 0.006, SVcorr). A further significant increase in the BOLD signal was also observed in the left lateral occipital cortex (*x* = −46; *y* = −70; *z* = −8; *k* = 141 voxels; Z = 4.38; *p* < 0.05, WBcorr) in a likely location of the extrastriate body area (EBA; Downing et al., 2001).

We also found activations in two regions not typically found to be active in previous RHI studies. One significant cluster of active voxels spanned the left posterior cingulate and retrosplenial cortex (*x* = –2, *y* = –48, *z* = 10; *k* = 216 voxels; Z = 4.04; *p* < 0.05, WBcorr), and another significant activation was located in the left dorsolateral prefrontal cortex (dlPFC; *x* = – 24, *y* = 22, *z* = 50; cluster = 216 voxels; Z = 4.54; *p* < 0.05, WBcorr).

In Extended Data Table 2-1, we report all activations in the whole-brain space at *p* < 0.001 uncorrected for multiple comparisons in a purely descriptive approach. For those with a particular interest in the previous fMRI literature on the RHI (see Introduction), it is worth mentioning that activation peaks that did not survive correction for multiple comparisons were also observed in the left putamen (*p* < 0.001, uncorrected), right cerebellum (*p* < 0.001, uncorrected), and right supramarginal cortex (*p* < 0.001, uncorrected); these regions have previously been associated with the RHI. Activation peaks did not survive in the insular cortex, another candidate region.

#### BCI model and parametric modulation

**Figure 5.**
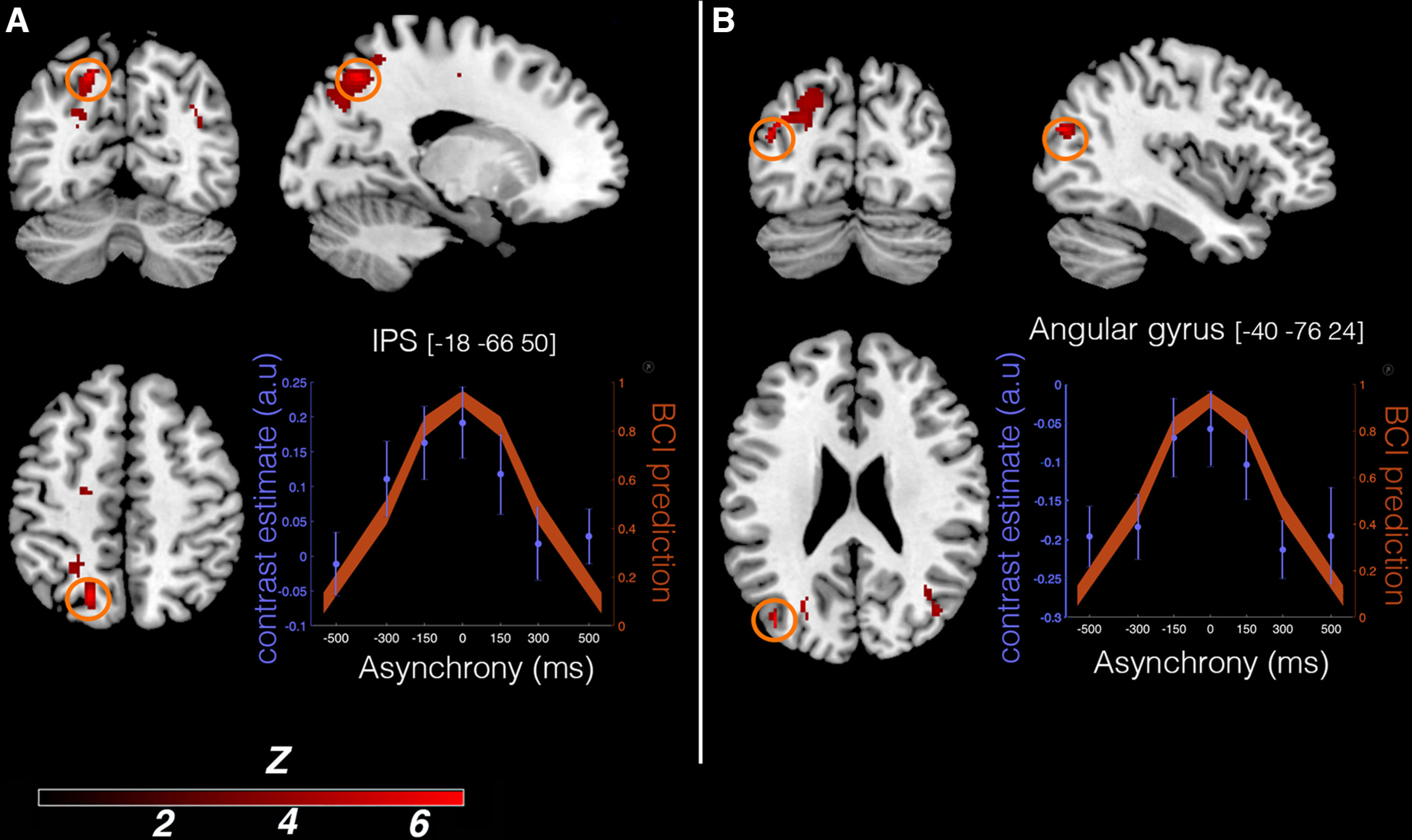
Activity in the PPC reflects individual BCI model predictions. The level of activity in the PPC is positively linearly related to the probability of the emergence of the rubber hand illusion as predicted by our BCI model, as observed in the parametrical modulation analysis. ***A***, ***B***, Two significant peaks of activation are displayed (*p* < 0.05, WBcorr), one located in the left angular gyrus (*x* = −40, *y* = −76, *z* = 24; ***A***) and one in the left IPS (*x* = −18, *y* = −66, *z* = 50; ***B***). For display purposes only, the activation map is displayed at a threshold of *p* < 0.001 (uncorrected for multiple comparisons; cluster threshold, 10 voxels), projected on a single-subject T1 MNI template (Fig. 6, presentation on the participants' mean structural MRI). The plots display the mean BOLD signal level (±SEM; blue dots and axis) in the respective region (left angular gyrus, left plot; left IPS, right plot) and mean BCI model prediction (orange shape and axis) as a function of visuotactile asynchrony. (Note that these mean BCI model plots across the whole sample are for illustration purposes only; the analysis was conducted at the first level with a parametric modulator specific to each participant's individual behavioral profile. See Materials and Methods for details.)

**Figure 6.**
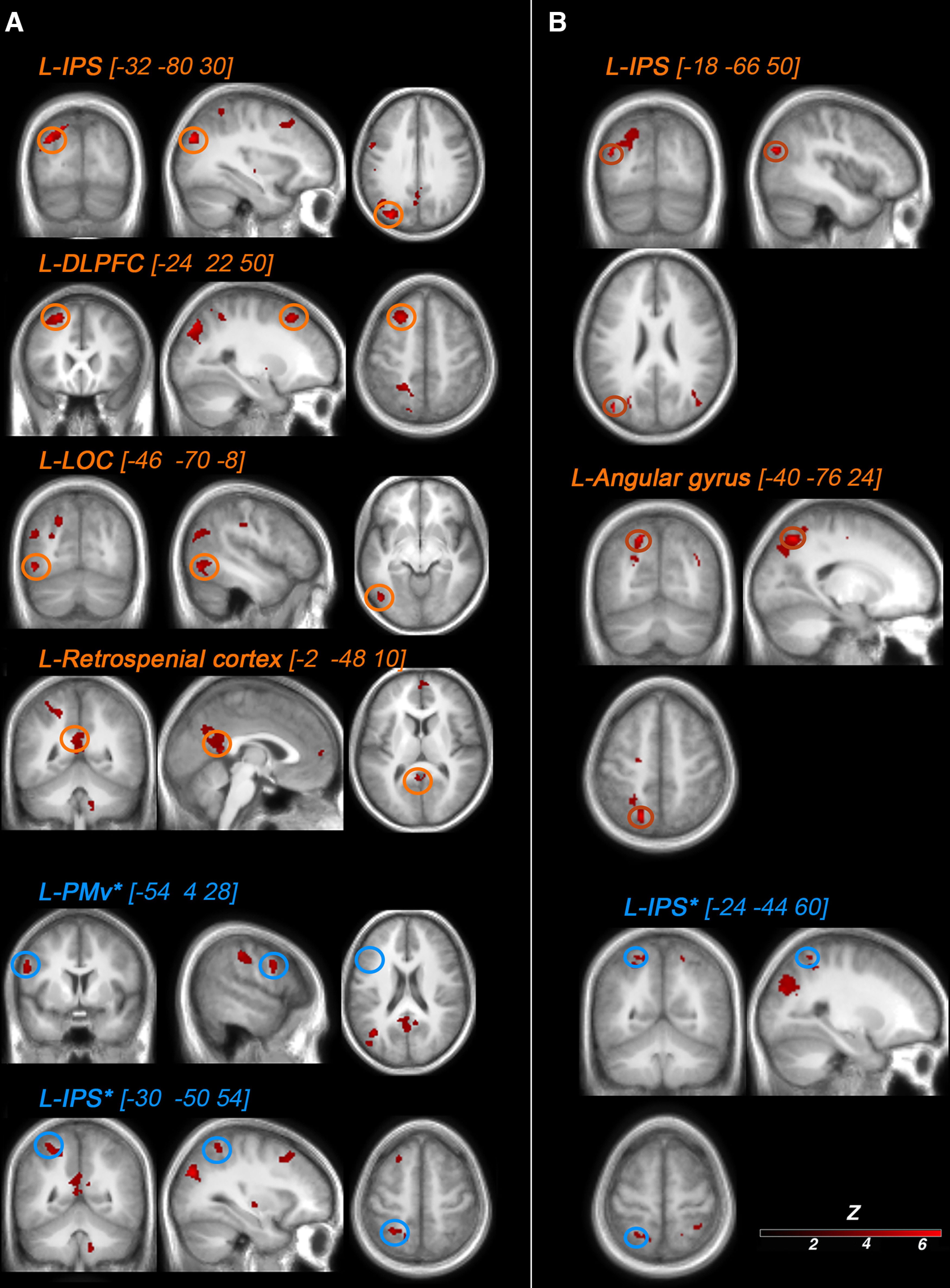
***A***, ***B***, Activations related to RHI detection (***A***) and predictions of the BCI model (***B***) are presented on a mean T1-weighted MRI from the current group of participants for more precise anatomic localization. For information about the contrasts and the statistical thresholds used for the activation maps, see Figure 4 for ***A*** and Figure 5 for ***B***.

In the second major analysis, we looked for neural responses that were predicted by the BCI model based on each participant's individual response profile (i.e., variations in BOLD signal that were linearly related to the probability of illusion emergence as predicted by the model; Figs. 5, 6*B*, Table 2). In line with our hypothesis, we found a large significant cluster of active voxels located in the PPC (*p* = 0.001, WBcorr; *k* = 621 voxels; *t* = 6.43; Z = 5.03), with two individual peaks with this cluster that survived the peak-height test: one peak located in the most posterior part of the left angular gyrus (*x* = −40, *y* = −76, *z* = 24; Z = 5.03; *p* = 0.016, WBcorr) and another significant peak located in the posterior part of the left IPS (*x* = −18, *y* = −66, *z* = 50; Z = 4.98; *p* = 0.019, WBcorr). In line with our anatomic hypothesis (Ehrsson et al., 2004), we also observed significant activation in a second section of the left IPS, located in the middle part of this sulcus (*x* = −24, *y* = −54, *z* = 60; Z score = 3.34; *p* = 0.035, SVcorr). However, no significant activation was observed in the PMv, not even at the descriptive threshold of *p* < 0.001 uncorrected, contrary to what we had hypothesized (Extended Data Table 3-1).

**Table 2.** Activations significantly related to the probability of emergence of the rubber hand illusion as predicted by our BCI model

MNI coordinates (mm)			Cluster level		Peak level	Anatomical region (functional area)
*x*	*y*	*z*	Cluster size (k)	*p* (FWE)	*z*	
−40	−76	24	621	0.016	5.03	L angular gyrus
−18	−66	50		0.019	4.48	L IPS
−26	−54	60		0.035	3.34	L IPS*

L, Left.

*Small-volume correction.

10.1523/JNEUROSCI.0656-22.2022.t3-1 Table 3-1 Table of results for the parametrical modulation analysis (*p* < 0.001 uncorrected; extent threshold = 10 voxels). Download Table 3-1, DOCX file.

## Discussion

We used an RHI detection task to investigate the neural basis of perceived body ownership, accounting for trial-to-trial variability in illusion elicitations, and fitting fMRI responses to a BCI model of multisensory perception. There were two main findings. First, we observed increased activity in the PPC (left IPS) and premotor cortex (left PMv) when participants felt the rubber hand was their own compared with when it did not, suggesting a link between multisensory integration in these areas during the critical period of stimulation leading up to illusion elicitation and perceptual changes in body ownership. Second, our BCI model predicted the probability of the RHI emergence based on activity in the left PPC. This suggests that the PPC implements the causal inference of body ownership, which advances understanding of the computational role of this region in multisensory own-body perception.

### PPC implements BCI of body ownership

The PPC is critical for multisensory processing (Berlucchi and Vallar, 2018). PPC activity reflects the integration of somatosensory and visual signals (Grefkes and Fink, 2005; Kavounoudias et al., 2008; Beauchamp et al., 2010; Gentile et al., 2011, 2013; Petkova et al., 2011; Brozzoli et al., 2012) and electrophysiological studies in nonhuman primates show convergence of signals from visual and somatosensory primary cortices in the PPC, highlighting its role in multisensory processing at the single-neuron and neuronal population levels (Duhamel et al., 1998; Graziano et al., 2000; Avillac et al., 2004, 2007; Whitlock, 2017). IPS activation is consistently identified in RHI studies (Ehrsson et al., 2004; Ehrsson, 2005; Gentile et al., 2013; Limanowski and Blankenburg, 2016b) during the relatively stable period after the illusion had already begun (∼10–45 s of stimulation) when contrasting an illusion condition with temporally and spatially congruent stimulations against control conditions that grossly violate congruence rules (Ehrsson et al., 2004; Ehrsson, 2005; Petkova et al., 2011; Gentile et al., 2013). However, in previous studies the illusion was not quantified on a trial-by-trial basis (i.e., participants passively experienced the illusion without a detection or rating task), so a tight link between changes in neural activity and perceptual changes in body ownership could not be established. In contrast, the current results imply that multisensory integration is the causal mechanism for the RHI by showing that the neural activity reflecting subtle changes in visuosomatosensory integration in the PPC, and premotor cortex (see below), during the critical period leading up to illusion-elicitation coincide with the emergence of subjective hand ownership on a trial-by-trial-basis.

Our findings provide insights into the possible neural computations occurring in the PPC during own-body perception. Specifically, activity at two foci in the left IPS and posterior angular gyrus varies with the probability of emergence of the RHI as predicted by our BCI model. We found an activation peak in the middle of the IPS consistent with a previous RHI study (Ehrsson et al., 2004) and reports that neuronal populations here integrate visual and tactile signals (Colby and Duhamel, 1991; Duhamel et al., 1998; Huang et al., 2012). The second IPS peak was located more posteriorly in a site associated with visuotactile integration of hand signals in peripersonal space (Lloyd et al., 2003; Makin et al., 2007; Brozzoli et al., 2011). In the same cluster, we observed an activation peak in the posterior angular gyrus associated with multisensory integration and visuospatial representations of the upper limb (Vingerhoets, 2014). Our findings suggest that the PPC dynamically infers the most likely causal structure of different sensory streams of events and postural states based on temporal and spatial correlations and prior perceptual experiences, which determine the extent to which sensory signals should be fused (RHI) or segregated (no RHI). We extend BCI principles from previous neuroimaging work on multisensory perception of audiovisual information (Rohe and Noppeney, 2015, 2016; Cao et al., 2019), which propose that BCI estimates are implemented by the PPC. Although the specific neuronal populations mediating own-body perception and audioviusal perception in the PPC probably differ, our findings suggest that similar BCI principles may distinguish self from the external world in the PPC, which is relevant for theories of probabilistic Bayesian causal inference as a unifying neuroscience theory (Shams and Beierholm, 2021).

### Premotor cortex and body ownership

PMv activity reflected positive judgments of hand ownership on a trial-by-trial level across the different levels of visuotactile delays. This finding extends previous fMRI RHI studies (Ehrsson et al., 2004; Tsakiris et al., 2007; Gentile et al., 2013; Bekrater-Bodmann et al., 2014; Limanowski and Blankenburg, 2016b; Grivaz et al., 2017) by revealing a link between visuosomatosensory integration in this area and the perceptual elicitation of the RHI. The involvement of the PMv in multisensory perception of one's own body is consistent with a role in integrating visual, tactile and proprioceptive signals from the upper limb in humans (Lloyd et al., 2003; Gentile et al., 2011) and primates (Rizzolatti et al., 1981a,b; Graziano et al., 1997; Fogassi et al., 1999; Graziano, 1999; Graziano and Gandhi, 2000), and previous fMRI studies that have reported correlations between individual differences in the strength of the RHI, as rated by questionnaires, and the amplitude of the RHI-associated activation in this region (Ehrsson et al., 2004; Ehrsson, 2005; Gentile et al., 2013). However, the basis for individual differences in the RHI is unclear and likely mediated by factors aside from multisensory integration. Thus, the current findings provide more compelling evidence for a link between body ownership and activity in the PMv as the relationship was established within subjects on a trial-by-trial basis and driven by subtle changes in degrees of visuotactile asynchrony in line with a multisensory theory of body ownership (Ehrsson et al., 2004; Samad et al., 2015; Ehrsson, 2020).

PMv activity did not significantly fit BCI model predictions unlike that hypothesized. In contrast, Fang et al. (2019) applied a BCI model to a reaching behavior in monkeys as a proxy for body ownership (pointing toward a target after the induction of an illusion similar to the RHI) and found that activity in the premotor cortex neurons matched the BCI model (for review, see Ehrsson and Chancel, 2019). However, the arm-ownership illusion was induced using a visuoproprioceptive spatial conflict, not a visuotactile temporal conflict. Thus, we speculate that the PMv may be more involved in visuoproprioceptive causal inference based on spatial correspondences than on visuotactile temporal correspondences (Graziano, 1999; Limanowski and Blankenburg, 2016a), although the indirect behavioral evidence and neurophysiological evidence in monkeys are different from our methods, making direct comparisons difficult. These considerations notwithstanding, there are changes in effective connectivity between the PPC and the PMv during the RHI (Gentile et al., 2013; Limanowski and Blankenburg, 2015; Casula et al., 2022), which are anatomically connected, that may vary with the level of sensory uncertainty and the prior (e.g., experimental context) magnitude/type of multisensory conflict. The neural implementation of multisensory BCI may have a hierarchical organization (Rohe and Noppeney, 2015, 2016; Cao et al., 2019; Rohe et al., 2019) where parietal regions implement multisensory estimates of body ownership, regardless of the context (Ganguli et al., 2008; Suzuki and Gottlieb, 2013), and frontal regions integrate contextual cues, and prior expectations, taking into account sensory uncertainty (Gau and Noppeney, 2016; Kayser and Kayser, 2018). We speculate that the premotor cortex maintains an updated internal representation of the multisensory/postural state of the own hand, implementing context and priors for the multisensory estimation process in the PPC in a dynamic process that involves top-down and bottom-up interactions between these two regions. Future studies should manipulate the level of sensory noise/uncertainty and priors to further understand how the premotor and posterior parietal cortices cooperate to govern body ownership.

### RHI detection activity in prefrontal and posterior cingulate areas

When the RHI was reported, increased activity was observed in the left lateral occipital cortex in a likely location of the EBA (Downing et al., 2001). This part of the lateral occipital cortex is known to show cross-modal effects and is activated during the RHI and similar illusions (Gentile et al., 2013; Guterstam et al., 2013; Limanowski et al., 2014; Limanowski and Blankenburg, 2015, 2016b). We also observed increased activation in the left posterior cingulate and retrosplenial cortex, which are not usually reported in RHI studies with passive participants. We propose that these neural responses relate to the process when the feeling of the RHI is translated into the concepts of “my hand” versus “not my hand” in the ownership detection task. Medial posterior regions are related to self-referential processing (e.g., self-name and self-face; Northoff and Bermpohl, 2004; Northoff et al., 2006), awareness of self-related information (Tacikowski et al., 2017), and self-concept (Qin and Northoff, 2011; Tacikowski et al., 2017). Thus, illusory changes in bodily self-perception during the RHI could have led to transient changes in self-concept when participants completed the explicit ownership judgments. However, this hypothesis should be directly tested in future studies. Moreover, we suggest that increased activity in the dlPFC corresponds to more general decisional processes in the detection task (Kim and Shadlen, 1999; Miller and Cohen, 2001; Carter and van Veen, 2007; Hanks et al., 2015; Jamali et al., 2019) because the dlPFC is typically not related to self-referential processing (Tacikowski et al., 2017).
