# Supplementary material for: Causal Inference of Body Ownership in the Posterior Parietal Cortex
Source: J Neurosci. 2022 Sep 14;42(37):7131–43. doi: 10.1523/JNEUROSCI.0656-22.2022 (PMC9480881; doi:10.1523/JNEUROSCI.0656-22.2022)
Supplement: Table 1-1 — Individual estimated parameters for the BCI model. Download Table 1-1, DOCX file. [file ns-JN-RM-0656-22-s01.docx]

| Participant | *p*_same_ | σ | λ | Negative log-likelihood |
| --- | --- | --- | --- | --- |
| 1 | 0.26 | 94 | 3.5 x 10^-11^ | 38 |
| 2 | 0.89 | 123 | 2.5 x 10^-11^ | 48 |
| 3 | 0.51 | 233 | 6.5 x 10^-5^ | 78 |
| 4 | 0.52 | 141 | 7.2 x 10^-11^ | 59 |
| 5 | 0.70 | 209 | 7.2 x 10^-7^ | 66 |
| 6 | 0.74 | 492 | 5.7 x 10^-6^ | 13 |
| 7 | 0.30 | 74 | 5.2 x 10^-1^ | 82 |
| 8 | 0.42 | 206 | 8.5 x 10^-7^ | 75 |
| 9 | 1.00 | 74 | 5.0 x 10^-9^ | 32 |
| 10 | 0.44 | 248 | 5.0 x 10^-6^ | 33 |
| 11 | 0.42 | 258 | 6.5 x 10^-5^ | 83 |
| 12 | 0.44 | 167 | 5.0 x 10^-9^ | 69 |
| 13 | 0.92 | 118 | 2.3 x 10^-1^ | 70 |
| 14 | 0.78 | 92 | 9.2 x 10^-2^ | 56 |
| 15 | 0.54 | 252 | 7.3 x 10^-6^ | 81 |
| 16 | 0.81 | 177 | 5.2 x 10^-1^ | 89 |
| 17 | 0.90 | 89 | 5.9 x 10^-2^ | 50 |
| 18 | 0.99 | 192 | 1.1 x 10^-9^ | 22 |
| 19 | 0.96 | 126 | 8.3 x 10^-12^ | 47 |
| 20 | 0.62 | 249 | 1.8 x 10^-10^ | 73 |
| 21 | 0.32 | 154 | 1.4 x 10^-7^ | 54 |
| 22 | 1.00 | 72 | 2.9 x 10^-2^ | 28 |
| 23 | 0.82 | 143 | 1.9 x 10^-1^ | 71 |
| 24 | 0.38 | 149 | 3.8 x 10^-10^ | 61 |
| 25 | 0.96 | 74 | 3.0 x 10^-2^ | 40 |
| 26 | 0.73 | 168 | 1.2 x 10^-6^ | 63 |
| 27 | 1.00 | 108 | 3.5 x 10^-2^ | 31 |
| 28 | 0.93 | 64 | 2.2 x 10^-16^ | 30 |
| 29 | 0.29 | 156 | 3.5 x 10^-7^ | 39 |
| 30 | 0.96 | 81 | 4.9 x 10^-8^ | 36 |
| *Mean* | *0.69* | *159* | *0.06* | *54* |
| *SEM* | *0.05* | *15.7* | *0.02* | *3.7* |

Extended Data Table 1: Individual estimated parameters for the BCI model
