# Supplementary material for: Causal Inference of Body Ownership in the Posterior Parietal Cortex
Source: J Neurosci. 2022 Sep 14;42(37):7131–43. doi: 10.1523/JNEUROSCI.0656-22.2022 (PMC9480881; doi:10.1523/JNEUROSCI.0656-22.2022)
Supplement: Table 2-1 — Result table for the answer-based contrasts (p < 0.001 uncorrected; extent threshold = 10 voxels), localization is based on peak activity. Download Table 2-1, DOCX file. [file ns-JN-RM-0656-22-s02.docx]

| **“Yes” > “No”** | | | | | | | | | | | | |
| --- | --- | --- | --- | --- | --- | --- | --- | --- | --- | --- | --- | --- |
| MNI coordinate (mm) | | | Cluster - level | | | | Peak - level | | | | |  |
| x | y | z | *p*_FWE_ | *q*_FDR_ | k_E_ | *p*_uncorr_ | *p*_FWE_ | *q*_FDR_ | T | Z | *p*_uncorr_ | *Anatomical localization* |
| -32 | -80 | 30 | 0.00 | 0.00 | 693 | 0.00 | 0.01 | 0.11 | 6.62 | 5.09 | 0.00 | L - IPS |
| -24 | 22 | 50 | 0.01 | 0.01 | 216 | 0.00 | 0.12 | 0.28 | 5.59 | 4.54 | 0.00 | L – superior frontal sulcus (PF dlc) |
| -46 | -70 | -8 | 0.05 | 0.04 | 141 | 0.00 | 0.21 | 0.38 | 5.32 | 4.38 | 0.00 | L – inferior occipital sulcus (LOC) |
| -56 | -28 | 42 | 0.06 | 0.04 | 136 | 0.01 | 0.40 | 0.44 | 4.97 | 4.17 | 0.00 | L – post central sulcus |
| -28 | -14 | -2 | 0.71 | 0.53 | 37 | 0.11 | 0.45 | 0.44 | 4.90 | 4.13 | 0.00 | L - putamen |
| -20 | -48 | 50 | 0.02 | 0.02 | 178 | 0.00 | 0.48 | 0.44 | 4.86 | 4.10 | 0.00 | L – superior parietal gyrus |
| -2 | -48 | 10 | 0.00 | 0.00 | 313 | 0.00 | 0.54 | 0.44 | 4.77 | 4.04 | 0.00 | L –cingulate gyrus / retrosplenial Co |
| 10 | -52 | -48 | 0.47 | 0.30 | 55 | 0.06 | 0.59 | 0.44 | 4.71 | 4.01 | 0.00 | R- cerebellum |
| -54 | 4 | 28 | 0.41 | 0.28 | 60 | 0.05 | 0.67 | 0.48 | 4.61 | 3.94 | 0.00 | L – precentral gyrus (PMv) |
| -10 | 6 | 18 | 0.98 | 0.82 | 12 | 0.35 | 0.71 | 0.48 | 4.55 | 3.90 | 0.00 | L – caudate nucleus |
| 12 | -44 | -14 | 0.98 | 0.82 | 13 | 0.33 | 0.77 | 0.49 | 4.46 | 3.85 | 0.00 | R – cerebellum |
| 22 | -6 | 50 | 0.95 | 0.82 | 17 | 0.27 | 0.79 | 0.49 | 4.44 | 3.83 | 0.00 | R –superior frontal sulcus |
| -20 | 62 | 14 | 0.40 | 0.28 | 61 | 0.04 | 0.89 | 0.53 | 4.27 | 3.72 | 0.00 | L – middle frontal gyrus |
| -2 | -24 | 42 | 0.99 | 0.82 | 10 | 0.39 | 0.90 | 0.53 | 4.26 | 3.71 | 0.00 | L –cingulate sulcus |
| 8 | 52 | 6 | 0.18 | 0.13 | 91 | 0.02 | 0.94 | 0.59 | 4.15 | 3.63 | 0.00 | R -medial superior frontal gyrus |
| -24 | -16 | 46 | 0.97 | 0.82 | 14 | 0.31 | 0.97 | 0.68 | 4.03 | 3.55 | 0.00 | L – precentral gyrus |
| 10 | -52 | 18 | 0.87 | 0.69 | 25 | 0.18 | 0.98 | 0.68 | 4.01 | 3.53 | 0.00 | R –cingulate gyrus |
| -24 | -8 | 10 | 0.99 | 0.82 | 11 | 0.37 | 0.99 | 0.72 | 3.89 | 3.45 | 0.00 | L - Putamen |
| -18 | 40 | 46 | 0.81 | 0.60 | 30 | 0.14 | 0.99 | 0.75 | 3.85 | 3.42 | 0.00 | L – sup frontal gyrus |
| -6 | -38 | 46 | 0.95 | 0.82 | 18 | 0.25 | 1.00 | 0.76 | 3.80 | 3.39 | 0.00 | L –cingulate Sulcus |
| -18 | 48 | 34 | 0.78 | 0.59 | 32 | 0.13 | 1.00 | 0.77 | 3.76 | 3.36 | 0.00 | L –sup frontal gyrus |
| **“Yes” < “No”** | | | | | | | | | | | |  |
| MNI coordinate (mm) | | | Cluster - level | | | | Peak - level | | | | |  |
| x | y | z | *p*_FWE_ | *q*_FDR_ | k_E_ | *p*_uncorr_ | *p*_FWE_ | *q*_FDR_ | T | Z | *p*_uncorr_ |  |
| -10 | -94 | 22 | 0.15 | 0.04 | 99 | 0.01 | 0.06 | 0.06 | 5.91 | 4.72 | 0.00 | L - cuneus |
| 16 | -92 | 22 | 0.11 | 0.04 | 111 | 0.01 | 0.45 | 0.28 | 4.90 | 4.13 | 0.00 | R - cuneus |
| 16 | -84 | 6 | 0.29 | 0.06 | 73 | 0.03 | 0.89 | 0.42 | 4.28 | 3.72 | 0.00 | R – calcarine sulcus |
| 58 | -36 | 42 | 0.99 | 0.59 | 10 | 0.39 | 1.00 | 0.68 | 3.67 | 3.29 | 0.00 | R - SMG |

Extended Data Table 2: Result table for the answer-based contrasts (p < .001 uncorrected, extent threshold = 10 voxels), localization is based on peak activity.
