# Supplementary material for: Causal Inference of Body Ownership in the Posterior Parietal Cortex
Source: J Neurosci. 2022 Sep 14;42(37):7131–43. doi: 10.1523/JNEUROSCI.0656-22.2022 (PMC9480881; doi:10.1523/JNEUROSCI.0656-22.2022)
Supplement: Table 3-1 — Table of results for the parametrical modulation analysis (p < 0.001 uncorrected; extent threshold = 10 voxels). Download Table 3-1, DOCX file. [file ns-JN-RM-0656-22-s03.docx]

| **BCI modulation** | | | | | | | | | | | | |
| --- | --- | --- | --- | --- | --- | --- | --- | --- | --- | --- | --- | --- |
| MNI coordinate (mm) | | | Cluster - level | | | | Peak - level | | | | |  |
| x | y | z | *p*_FWE_ | *q*_FDR_ | k_E_ | *p*_uncorr_ | *p*_FWE_ | *q*_FDR_ | T | Z | *p*_uncorr_ | *Anatomical localization* |
| -40 | -76 | 24 | 0.000 | 0.000 | 621 | 0.000 | 0.016 | 0.030 | 6.43 | 5.03 | -40 | L – angular gyrus |
| -20 | -14 | 52 | 0.975 | 0.829 | 13 | 0.354 | 0.829 | 0.566 | 4.31 | 3.76 | -20 | Junction L –pre-central sulcus/ sup frontal sulcus |
| -36 | 30 | 40 | 0.987 | 0.829 | 10 | 0.418 | 0.833 | 0.566 | 4.30 | 3.75 | -36 | L - middle frontal gyrus |
| 34 | -46 | 58 | 0.889 | 0.829 | 24 | 0.209 | 0.849 | 0.566 | 4.28 | 3.73 | 34 | R – superior parietal gyrus |
| 36 | -66 | 30 | 0.434 | 0.517 | 61 | 0.054 | 0.862 | 0.566 | 4.25 | 3.72 | 36 | R – angular gyrus |
| -48 | -20 | 34 | 0.944 | 0.829 | 18 | 0.275 | 0.953 | 0.634 | 4.04 | 3.57 | -48 | L - post central gyrus (close to post central sulcus) |
| 18 | -56 | 58 | 0.975 | 0.829 | 13 | 0.354 | 0.983 | 0.691 | 3.90 | 3.47 | 18 | R - superior parietal gyrus |

Extended Data Table 3: Result table for the parametrical modulation analysis (p<.001 uncorrected, extent threshold = 10 voxels).
